# Case Report: Prevention of Rhabdomyolysis-Associated Acute Kidney Injury by Extracorporeal Blood Purification With Cytosorb^®^

**DOI:** 10.3389/fped.2021.801807

**Published:** 2022-01-24

**Authors:** Simon Rauch, Andrea Borgato, Ewald Gruber, Carlo Leggieri, Matthias Bock, Paolo Mario Enrico Seraglio

**Affiliations:** ^1^Department of Anesthesia and Intensive Care Medicine, “F. Tappeiner” Hospital, Merano, Italy; ^2^Institute of Mountain Emergency Medicine, Eurac Research, Bolzano, Italy; ^3^Department of Surgery, “F. Tappeiner” Hospital, Merano, Italy; ^4^Department of Anaesthesiology, Perioperative Medicine and Intensive Care Medicine, Paracelsus Medical University, Salzburg, Austria

**Keywords:** rhabdomyolysis, crush injury, acute kidney injury, blood purification, Cytosorb^®^

## Abstract

Acute kidney injury (AKI) is a severe complication of rhabdomyolysis. The pathophysiology of rhabdomyolysis-associated AKI is complex, but myoglobin related damage plays a major role. Extracorporeal removal of myoglobin is therefore an appealing target to prevent AKI, however, attempts to remove myoglobin with standard dialysis membranes have so far been disappointing. Here we report the case of a 12-year-old boy with severe trauma-related rhabdomyolysis where we successfully utilized continuous renal replacement therapy in combination with Cytosorb^®^ to eliminate myoglobin and prevent AKI. The early use of extracorporeal myoglobin removal with Cytosorb^®^ after severe rhabdomyolysis might be an option and should be further investigated as a tool to prevent the development of AKI.

## Introduction

Rhabdomyolysis is a clinical syndrome caused by damage to skeletal muscle and release of its breakdown products into the circulation. Acute kidney injury (AKI) is a severe complication of rhabdomyolysis (1, 2) with an incidence in adults of about 30–50% after trauma-related rhabdomyolysis (3–5). In children the incidence (4, 6, 7) as well as the risk of fatal outcome (4) is lower. Most studies on the risk of and outcome after rhabdomyolysis-associated AKI in children were done after natural disasters, especially earthquakes.

The pathophysiology of rhabdomyolysis-associated AKI is complex, and different mechanisms such as tubular damage by oxidative injury, tubular obstruction by precipitated Tamm-Horsfall protein-myoglobin complexes, and renal vasoconstriction have been described (8–10). In most of these processes, myoglobin released by damaged muscle plays a major role and is directly involved (8, 13).

The mainstay of prevention and treatment of rhabdomyolysis-associated AKI is the early and aggressive hydration because patients with rhabdomyolysis are usually fluid depleted as water sequestrates in the injured muscle (8). Administration of bicarbonate with urine alkalinization reduces both tubular precipitation of the Tamm–Horsfall protein–myoglobin complex and oxidative tubular injury, and can be considered if urine pH is <6.5 (8). There is conflicting evidence regarding the administration of Mannitol, which might be administered to increase urinary output (8, 11). When severe AKI results in refractary hyperkalemia, acidosis and/or volume overload, renal replacement therapy is indicated (8).

Extracorporeal removal of myoglobin is an appealing target to prevent AKI, however, attempts to remove myoglobin with standard dialysis membranes have so far been unsuccessful (12). Here we report the case of a child with severe trauma-related rhabdomyolysis without established AKI where we initiated continuous renal replacement therapy combined with a Cytosorb^®^ cartridge as a measure to prevent rhabdomyolysis-associated AKI. The child's parents have given their written informed consent to publish the case.

## Case Report/Case Presentation

**Figure 1:**
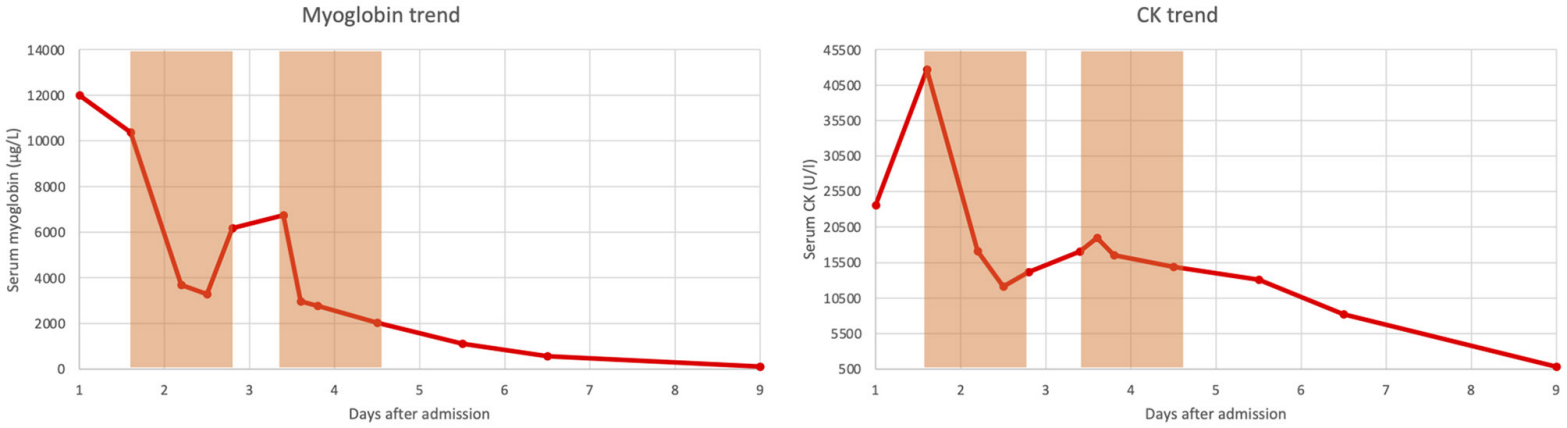
Trend of serum myoglobin and serum creatinine kinase (CK) over time. The light orange boxes denote the periods in which continuous veno-venous hemodiafiltration combined with Cytosorb^®^ was performed.

A 12-year-old boy was hit by a motorcycle while riding his bicycle. The motorcycle ran over the boy's legs causing an open wound in the right groin with a massive bleeding. Upon arrival of the ambulance and emergency physician the boy was in hemorrhagic shock. The bleeding was compressed manually, an intravenous access was obtained, 500 ml of crystalloid fluids and 500 mg of tranexamic acid were administered, and the patient was transported to our hospital. A contrast enhanced whole body CT scan was performed in the emergency department which revealed a dissection of the right common femoral artery and a laceration of the common femoral vein; peripheral pulses were not palpable. The patient was intubated and brought to the operating theater for vascular surgery; a femoral-femoral bypass with a saphenous graft was made and the common femoral vein, which was found to be completely disrupted, was ligated proximally and distally. A medial and lateral fasciotomy was performed at the lower leg to prevent a compartment syndrome. The time from the accident to leg reperfusion was estimated to be 3.5 h. Postoperatively the patient was admitted to the intensive care unit (ICU) and extubated after a few hours. On Doppler ultrasound a normal, triphasic flow profile was found down to the distal tibial and peroneal artery. Within the next hours a massive rhabdomyolysis developed with creatinine kinase (CK) and myoglobin values reaching a peak of >42,670 U/l (upper limit of laboratory detection) and >12,000 μg/l (upper limit of laboratory detection), respectively. Trend of CK and myoglobin over time is depicted in Figure 1. Balanced crystalloid fluids were administered intravenously to maintain a urinary output of about 200 ml per hours. Despite still normal values of serum creatinine and urea as well as preserved diuresis, we decided to initiate continuous veno-venous hemodiafiltration (CVVHDF) (PrisMax System, Baxter International Inc., USA) with a high flux filter (AN69, Baxter International Inc., USA) and to add a Cytosorb^®^ cartridge (CytoSorbents Europe GmbH, Germany) to the dialysis circuit. CVVHDF was set with a blood flow rate of 150 ml/min, dialysate flow rate of 500 ml/hour and substitution flow rate of 1,000 ml/h; regional anticoagulation with Citrate was used. Twelve hours after initiation of CVVHDF plus Cytosorb^®^, CK and myoglobin had substantially decreased (Figure 1), yet, during the subsequent 12 h of extracorporeal treatment an increase of both parameters was noted (Figure 1). After 24 h of CVVHDF plus Cytosorb^®^, extracorporeal treatment was interrupted for 16 h, but because CK and myoglobin continued to increase (Figure 1), a second cycle of CVVHDF plus Cytosorb^®^ was started and continued for another 24 h (Figure 1). This led again to a marked decrease in both, CK and myoglobin values. Serum creatinine and urea remained in the normal range also after termination of extracorporeal treatment and CK and myoglobin continued to decrease (Figure 1).

The length of stay in the ICU was 10 days, whereafter the patient was transferred to the surgical ward and after another 22 days he was discharged from hospital.

## Discussion/Conclusion

Rhabdomyolysis is a pathogenetic cause of AKI (1, 2), and myoglobin plays an essential role in the development of AKI (8, 13). Extracorporeal therapy is currently only recommended to replace the failing kidney function i.e., for treatment of established and severe AKI. Yet, there is a strong and logical rationale for quick and effective removal of myoglobin in acute rhabdomyolysis (12), which could prevent the development of AKI. Attempts to remove myoglobin with standard dialysis membranes have so far been disappointing. Reasons for this depend on the nature of the molecule, its distribution in the organism, on the mechanism of solute transport over and the structure of the membrane in the extracorporeal circuit (12). Therefore, alternative techniques have been investigated. The use of super high-flux or high cut-off membranes are more effective in removing myoglobin than standard high flux membranes, however, excessive albumin loss is a major concern (14–16). The adoption of the Cytosorb^®^ (CytoSorbents Europe GmbH, Germany) cartridge is a novel approach for myoglobin removal. Cytosorb^®^ is a synthetic adsorption column composed of highly porous biocompatible polymer beads that are able to capture and absorb molecules smaller than 55 kDa (17). Primarily intended for the adsorption of cytokines in hyperinflammatory conditions (18), Cytosorb^®^ has a CE mark for the elimination of myoglobin since 2019. Myoglobin has a mass of 17 kD (19) and is therefore effectively adsorbed by Cytosorb^®^ (20). Also, Cytosorb^®^ has a much larger surface area (45,000 m^2^) than standard membranes used for continuous renal replacement therapy (about 1.8 m^2^) which contributes to a more efficient myoglobin removal. Some case reports and observational studies describe the successful utilization of Cytosorb^®^ for myoglobin removal in patients with established, rhabdomyolysis-associated AKI (9, 21–23). However, we are not aware of any report describing the preventive use of Cytosorb^®^ in severe rhabdomyolysis. Although the degree of CK and myoglobin elevation does not always predict the development of AKI (24, 25), we estimated a high risk of AKI development in our patient given the very high serum concentration of both molecules and decided for preventive CVVHDF in combination with Cytosorb^®^. Myoglobin values rapidly decreased after initiation of extracorporeal treatment. However, after 12 h, we noted an increasing trend. We supposed a rapid saturation of the absorber as already described (21) and, in fact, after Cytosorb^®^ replacement, myoglobin levels rapidly decreased again.

In conclusion, in patients with severe rhabdomyolysis, a preventive extracorporeal myoglobin removal with Cytosorb^®^ could be considered to impede the development of AKI.

## Data Availability Statement

The original contributions presented in the study are included in the article/supplementary material, further inquiries can be directed to the corresponding author.

## Ethics Statement

This study protocol was reviewed and approved by the ethics committee of the “Azienda Sanitaria dell'Alto Adige”, approval number 133–2021. Written informed consent was obtained from the child's parents for publication of the details of the medical case and any accompanying images. The research was conducted ethically in accordance with the World Medical Association Declaration of Helsinki.

## Author Contributions

SR and PMES: collected, retrieved, and analyzed data. SR: drafted the manuscript. All authors revised the manuscript for important intellectual content.

## Conflict of Interest

The authors declare that the research was conducted in the absence of any commercial or financial relationships that could be construed as a potential conflict of interest.

## Publisher's Note

All claims expressed in this article are solely those of the authors and do not necessarily represent those of their affiliated organizations, or those of the publisher, the editors and the reviewers. Any product that may be evaluated in this article, or claim that may be made by its manufacturer, is not guaranteed or endorsed by the publisher.
